# Diagnosis of vertebral osteomyelitis

**DOI:** 10.5194/jbji-7-23-2022

**Published:** 2022-01-27

**Authors:** Julian Maamari, Aaron J. Tande, Felix Diehn, Don Bambino Geno Tai, Elie F. Berbari

**Affiliations:** 1 Division of Infectious Diseases, Mayo Clinic, Rochester, MN, USA; 2 Department of Radiology, Mayo Clinic, Rochester, MN, USA

## Abstract

Native vertebral osteomyelitis (NVO) is a potentially fatal infection which has seen a gradual increase in its incidence over the past decades. The infection is insidious, presenting with symptoms of back pain. Fever is present in about 60 % of patients. Prompt diagnosis of NVO is important to prevent the development of complications. Numerous laboratory and imaging tools can be deployed to accurately establish the diagnosis. Imaging techniques such as magnetic resonance, nuclear imaging, and computed tomography are essential in diagnosing NVO but can also be useful in image-guided biopsies. Laboratory tools include routine blood tests, inflammatory markers, and routine culture techniques of aspirated specimens. Recent advances in molecular techniques can assist in identifying offending pathogen(s). In this review, we detail the arsenal of techniques that can be utilized to reach a diagnosis of NVO.

## 1 Introduction

Native vertebral osteomyelitis (NVO), also termed spondylodiscitis, is a potentially fatal condition that constitutes roughly 3 %–5 % of all osteomyelitis cases (Sobottke et al., 2008). Its incidence has increased from 2.9 cases to 5.4 cases per 100 000 people in the United States between 1998 and 2013, owing partly to a demographic shift towards an older and immunocompromised population (Issa et al., 2018). Due to relative rarity and nonspecific symptoms, delays in the diagnosis of NVO still happen despite the expanding use and availability of magnetic resonance imaging (MRI). A prospective study on NVO found a mean diagnostic delay of 45.5 d from the onset of symptoms (range 2–280 d). Other studies have suggested even longer delays, with variations attributed to the causative organism (Jean et al., 2017).

NVO is most commonly the result of hematogenous seeding of the avascular disc. Other causes include contiguous spread and direct inoculation during surgery (Zimmerli, 2010). The most common symptom at the time of presentation is back pain (Mylona et al., 2009). Although highly sensitive (86 %), this symptom lacks specificity, particularly among older adults. Other symptoms of NVO, such as fever (60 %) and neurologic deficits, including radiculopathy, urinary retention, limb weakness, paralysis, dysesthesia, or sensory loss (34 %) are less common (Mylona et al., 2009). Routinely performed inflammatory markers such as erythrocyte sedimentation rate (ESR) and C-reactive protein (CRP) are sensitive but also lack specificity (Zimmerli, 2010). Therefore, maintaining a high index of suspicion is crucial for establishing the diagnosis of NVO.

There are no widely agreed upon diagnostic criteria for diagnosing NVO, particularly in cases with negative blood and biopsy cultures. Instead, NVO is diagnosed through a compatible overall clinical picture, combined with suggestive imaging and laboratory findings (Berbari et al., 2015). Early diagnosis and treatment are essential to decrease the risk of complications, neurologic deficits, and mortality (Gupta et al., 2014). This review summarizes the literature on the various diagnostic modalities employed to diagnose NVO.

## 2 Laboratory studies

Inflammatory biomarkers, such as erythrocyte sedimentation rate (ESR) and C-reactive protein (CRP), are the most well-studied screening tests for NVO in the setting of back pain. (Berbari et al., 2015). Both markers have been found to have a sensitivity in the range of 94 %–100 %, particularly when used in combination (Berbari et al., 2015). Logistic regression of a cohort of 72 patients with suspected NVO undergoing image-guided biopsy revealed that the combination of ESR, CRP, and the presence of fever has the highest area under the curve (AUC = 0.72) for predicting a diagnosis of NVO. Enhancement of the predictive yield was observed when MRI results were factored in (Kihira et al., 2020). ESR is typically more elevated in common bacterial NVO than in tuberculous NVO, with more than 91 % of NVO patients having an initial ESR value > 50 mm h ${}^{-1}$ (Waheed et al., 2019). One study suggested that using a score that encompasses CRP, pain severity grading, and imaging findings may be a useful tool in the diagnosis, treatment, and follow-up of patients with NVO (Homagk et al., 2019). CRP and ESR may also help predict relapse following treatment (Ahn et al., 2020; McHenry et al., 2002; Chiang et al., 2019; Carragee et al., 1997). Serum white blood cell (WBC) count has low sensitivity and specificity. Leukocytosis is often absent or only mildly elevated (An and Seldomridge, 2006). Apart from CRP and ESR, no novel biomarkers have paved their way into clinical practice in recent decades. Efforts to identify other reliable biomarkers are warranted, especially in the setting of partially treated NVO or infection with an indolent organism.

## 3 Imaging modalities

Although MRI is the preferred imaging modality for the diagnosis of NVO, we recommend obtaining a plain radiograph of the spine as an initial test (Diehn, 2012). Plain radiography has low sensitivity at the early stages of the disease, but it may help identify other causes of back pain and establish spinal enumeration. Subtle findings, such as loss of definition, erosions, and irregularity of the vertebral end plates, typically lag behind the disease, only appearing 2 to 8 weeks after the onset of symptoms (Govender, 2005). If present on a prior radiograph, the disappearance of a previously seen degenerative gas in the disc space (disc space vacuum phenomenon) can be suggestive of NVO, particularly if it is associated with disc space widening and/or end plate erosions.

MRI is the preferred imaging modality for diagnosing NVO (Diehn, 2012). The sensitivity, specificity, and accuracy of MRI in diagnosing NVO are estimated at 97 %, 92 %, and 94 %, respectively (Table 1; Modic et al., 1985). MRI should ideally be performed with intravenous gadolinium contrast. It increases the sensitivity and specificity of the MRI, including a better depiction of a possible extension of infection to the epidural and paravertebral spaces. T2-weighted and post-contrast T1-weighted images should be acquired with fat suppression. A hallmark of the disease is the presence of marrow-replacing signal abnormalities, seen best on T1-weighted non-contrast images. The normal marrow is hyperintense compared with the intervertebral discs, whereas abnormal marrow is relatively hypointense. Such an abnormal marrow signal on T1-weighted images typically correlates with T2 hyperintensity, which is best seen on fat-suppressed T2-weighted images, and enhancement, which is best seen on post-contrast fat-suppressed T1-weighted images. (Berbari et al., 2015; Prodi et al., 2016). The disc itself may also be abnormally T2 hyperintense or enhancing. Although MRI can detect bone marrow edema as early as 48 h after disease onset, early findings may be nonspecific or atypical; the primary confounders are active sub-end plate degenerative changes (so-called Modic type I changes). In these patients, an MRI can be repeated in 2–4 weeks to further evaluate the diagnosis of NVO (Kamiya et al., 2019). The inclusion of diffusion-weighted imaging on MRI is sometimes used to help increase the specificity of bone marrow edema for NVO (Patel et al., 2014). Routine follow-up MRI for clinically improving patients on treatment is unnecessary, as the imaging resolution can lag behind clinical improvement (Kowalski et al., 2007). At times, MRI may provide clues to the causative organism (Hong et al., 2009); for example, a multilevel process with subligamentous extension and prominent paraspinal component with relative sparing of the disc spaces may suggest *Mycobacterium tuberculosis*.

**Table 1:** Sensitivity and specificity of CT scan and MRI in the detection of vertebral osteomyelitis.

Study authors	Year	CT scan			MRI	
		Sensitivity	Specificity	Study type	Sensitivity	Specificity
Modic et al.	1985	–	–	–	96 %	92 %
Osenbach et al.	1990	100 %	Could not assess	–	100 %	Could not assess
Bateman and Pevzner	1995	92 %	Could not assess	–	86 %	Could not assess
Torda et al.	1995	84 %	Could not assess	–	100 %	Could not assess
Dagirmanjian et al.	1996	–	–	–	95 %	Could not assess
Carragee et al.	1997	–	–	–	53 %	Could not assess
Chelsom and Solberg	1998	88 %	Could not assess	–	100 %	Could not assess
Fernandez et al.	2000	–	–	–	95 %	Could not assess
Love et al.	2000	–	–	–	91 %	77 %
Nolla et al.	2002	100 %	Could not assess	–	100 %	Could not assess
Gratz et al.	2002	100 %	87 %	PET/CT	100 %	85 %
McHenry et al.	2002	–	–	–	74 %	Could not assess
Ledermann et al.	2003	–	–	–	100 %	Could not assess
Zarrouk et al.	2006	–	–	–	100 %	Could not assess
Fuster et al.	2012	89 %	88 %	PET/CT	–	–
Nakahara et al.	2015	100 %	79 %	PET/CT	76 %	42 %
Smids et al.	2017	96 %	95 %	PET/CT	67 %	84 %
Tamm and Abele	2017	–	–	–	94 %	100 %
Kouijzer et al.	2018	100 %	83 %	PET/CT	100 %	92 %

Computed tomography (CT) is another imaging technique that can help diagnose NVO (Table 1). CT can be beneficial in cases where Modic type I changes are a primary consideration based on MRI, and the clinical findings do not strongly suggest an infection. In such patients, the absence of end plate cortical erosive changes makes NVO is less likely. CT is superior to MRI with respect to the evaluation of cortical bone and depicting the disc space vacuum phenomenon. In rare cases, gas in the disc is related to a gas-forming organism or other anatomic abnormality, such as a fistula with the gastrointestinal tract (Diehn, 2012).

Nuclear imaging techniques have also been employed successfully to diagnose NVO (Prodi et al., 2016). They may be the alternative in cases with severe degenerative arthritis, potential neuropathic arthropathy (Charcot spine), or when MRI is contraindicated (Love et al., 2000). Scintigraphy with single-photon emission computed tomography (SPECT) using Technetium-99m ( ${}^{\text{99m}}$ Tc) and Gallium-67 ( ${}^{67}$ Ga) tracers are the most widely used methods. Studies showed that ${}^{\text{99m}}$ Tc scintigraphy has high sensitivity (90 %) but moderate specificity. Combining the two techniques increases the sensitivity, with some studies suggesting that ${}^{67}$ Ga or ${}^{\text{99m}}$ Tc scanning alone may be insufficient to diagnose NVO. These studies demonstrated that these techniques were equivalent to MRI (Modic et al., 1985; Maurer et al., 1981; Hadjipavlou et al., 1998; Tamm and Abele, 2017). Combining both techniques is the standard of care if used in place of MRI (Tamm and Abele, 2017). Tracer uptake that is greater or anatomically discordant on the gallium (inflammation detecting) than on the technetium (metabolism detecting) portion of the combined nuclear medicine study is the finding which most strongly and accurately suggests NVO (Diehn, 2012). Positron emission tomography–computed tomography (PET/CT) has also been evaluated for the diagnosis of NVO. The literature suggests that the technique may be more accurate than combined ${}^{67}$ Ga and ${}^{\text{99m}}$ Tc scans with similar accuracy compared to MRI (Fuster et al., 2012; Kouijzer et al., 2018). The advantages of PET/CT include its superior spatial resolution and the better detection of metastatic infection. In addition, a CT scan itself may hold an advantage in detecting sequestra, cloacas, involucra, or intraosseous gas, which may form in chronic NVO (Pineda et al., 2009); however, MRI remains a superior imaging modality in detecting small intraspinal (e.g., epidural) and paraspinal abscesses (Tables 1, 2; Fuster et al., 2012; Kouijzer et al., 2018).

**Table 2:** Sensitivity and specificity of nuclear imaging techniques in the detection of vertebral osteomyelitis.

Nuclear imaging				
Study authors	Year	Sensitivity	Specificity	Comments
Bruschwein et al.	1980	90 %	85 %	Gallium bone scan
Maurer et al.	1981	92 %	94 %	Technetium bone scan; three-phase scan
Modic et al.	1985	91 %	78 %	Technetium bone scan
		93 %	Could not assess	Gallium bone scan
Osenbach et al.	1990	100 %	Could not assess	Technetium bone scan
Patzakis et al.	1991	100 %	Could not assess	Technetium bone scan
Nolla-Solé et al.	1992	90 %	Could not assess	Technetium bone scan
		100 %	Could not assess	Gallium bone scan
Lisbona et al.	1993	96 %	Could not assess	Technetium bone scan
		100 %	Could not assess	Gallium bone scan
Torda et al.	1995	87 %	Could not assess	Technetium bone scan
		100 %	Could not assess	Gallium bone scan
Bateman and Pevzner	1995	91 %	Could not assess	Technetium bone scan
		100 %	Could not assess	Gallium bone scan
Chelsom and Solberg	1998	85 %	Could not assess	Technetium bone scan
Hadjipavlou et al.	1998	100 %	100 %	Gallium bone scan
Gratz et al.	2000	93 %	Could not assess	Technetium bone scan; planar and SPECT
		81 %	Could not assess	Gallium bone scan; planar and SPECT
Love et al.	2000	82 %	23 %	Technetium bone scan; planar and SPECT
		36 %	92 %	Technetium bone scan (three phase)
		91 %	92 %	Gallium bone scan; planar and SPECT
Nolla et al.	2002	96 %	Could not assess	Technetium bone scan
		91 %	Could not assess	Gallium bone scan
Gratz et al.	2002	78 %	50 %	Technetium bone scan
		71 %	61 %	Gallium bone scan
Fuster et al.	2012	78 %	81 %	Gallium bone scan; combined with bone scan and SPECT
Tamm and Abele	2017	94 %	100 %	Gallium bone scan or technetium bone scan and SPECT

## 4 Biopsy methods and microbiology

**Figure 1:**
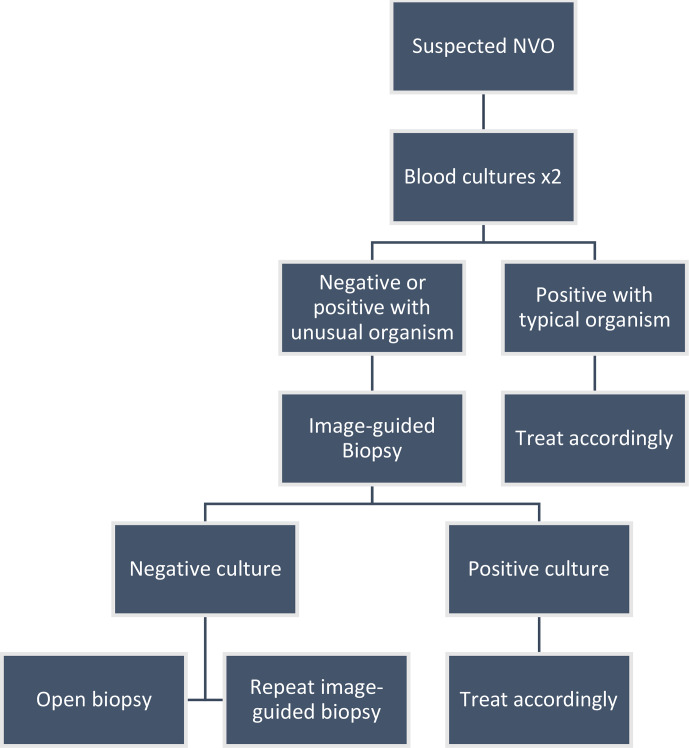
Approach to diagnosing a patient with native vertebral osteomyelitis.

Optimal management relies on the isolation of the causative organism. The initial step is collecting bacterial blood cultures, which are positive in approximately 58 % of cases (range 30 %–78 %) (Mylona et al., 2009; Zimmerli, 2010). The Infectious Diseases Society of America (IDSA) guidelines recommend obtaining two sets of bacterial blood cultures (aerobic and anaerobic) in patients with suspected NVO. When positive, blood cultures may obviate the need for biopsies (Berbari et al., 2015). However, the yield of blood cultures may be affected by previous antibiotic therapy. Most cases of NVO that result from hematogenous seeding are monomicrobial. Other causes associated with contiguous spread or direct inoculation tend to be more polymicrobial (Mavrogenis et al., 2017). If infection with a typical organism – i.e., *Staphylococcus aureus* complex, *Staphylococcus lugdunensis*, or *Brucella* species – is established with blood cultures or serologic testing, no further investigation may be necessary (Berbari et al., 2015). An image-guided biopsy is warranted when blood cultures or serologic testing does not establish the microbiologic diagnosis (Berbari et al., 2015). The two most widely recognized methods are image-guided percutaneous biopsy and open biopsy (McNamara et al., 2017). Percutaneous biopsies and aspirations are typically guided by CT or fluoroscopy (Kim et al., 2013). These sampling procedures can target the bone, disc, and adjacent infected spinal sites such as facet joints or paraspinal soft tissues, including abscesses. Intraspinal sampling (e.g., of epidural abscesses) can be performed if there are accessible dorsal, relatively large components to the intraspinal collections. Otherwise, it is not routinely performed due to the risk of inadvertent dural puncture. Percutaneous biopsies have variable microbiologic yields of 30.4 %–91 % (Chew and Kline, 2001; Pupaibool et al., 2015). Two meta-analyses calculated the cumulative yield between 48 % and 52 %, significantly lower than the 76 % yield in open biopsies (McNamara et al., 2017; Pupaibool et al., 2015). Factors that may increase the yield of the image-guided procedure include an elevated CRP; the use of a lower-gauge needle, increased number of specimens obtained; and, if present, the aspiration of a fluid collection (Husseini et al., 2020; Gras et al., 2014). The impact of prior antibiotic use on image-guided specimens' culture yield remains uncertain, and the findings of existing studies are conflicting: some studies indicate that prior antimicrobial therapy negatively impacted the yield, whereas some indicate no effect. The studies were limited in their retrospective design, sample size, and selection bias (Wong et al., 2021). If the initial biopsy is nondiagnostic, a second percutaneous biopsy may be warranted, although the exact increased yield is unclear (Gras et al., 2014). A repeat biopsy should be delayed at least 3 d after the initial biopsy, at which time the majority of positive cultures from the first should have resulted (Yeh et al., 2020). Alternatively, when the first image-guided biopsy is negative, it is reasonable to proceed with an open biopsy as the next step (Fig. 1; Berbari et al., 2015).

Specimens should be sent for both microbiologic and histopathologic examination. Histopathology reveals the presence of acute inflammatory cells in 69 %–95 % of cases (Iwata et al., 2019; Heyer et al., 2012). Biopsy specimens should be sent for aerobic and anaerobic bacterial cultures. Fungal, zoonotic, and mycobacterial etiologies should be considered in patients with culture-negative NVO, immunocompromising conditions, or risk factors such as living in endemic areas (Berbari et al., 2015; Mavrogenis et al., 2017). Patients who are immunocompromised are particularly susceptible to non-endemic fungal organisms such as *Candida* spp., *Aspergillus* spp., and *Cryptococcus neoformans* (Hong et al., 2009; Salaffi et al., 2021). *C. albicans* is responsible for more than half of candidal NVO cases, although *Nakaseomyces glabrata* – previously *C. glabrata* – is also becoming more common. Modern bacterial blood culture techniques are capable of identifying *Candida* species. Aspergillus NVO may mimic tuberculous NVO particularly when the intervertebral disc is spared, with the most commonly isolated species being *A. fumigatus* (Salaffi et al., 2021). In patients at risk of fungal infections, fungal serologies, antigen detection assays, and fungal blood cultures may also be useful (Berbari et al., 2015). Proving a diagnosis of NVO in these cases requires documenting a positive culture or histology result, a clinical picture compatible with NVO, and radiologic evidence of the infection (De Pauw et al., 2008). Coccidioidomycosis and blastomycosis are the most important endemic fungal infections that may cause NVO. *C. immitis* localizes to the bone in more than 50 % of diffuse cases, whereas bone involvement is noted in 14 %–60 % of diffuse blastomycosis, with the spine being the most commonly involved location (Salaffi et al., 2021; Hong et al., 2009). However, serologic testing for *Coccidioides* and *Blastomyces* species may be considered if epidemiologic factors exist (Berbari et al., 2015).

For *Brucella* NVO, serologies and *Brucella* blood cultures are diagnostic tests of choice. A cutoff of > 1 : 160 for *Brucella* antibodies or > 1 : 320 for the Coombs test is considered positive (Berbari et al., 2015; Tali et al., 2015). Pott's disease (tuberculous NVO) should be suspected among patients with known or suspected tuberculosis at another site or living in areas endemic for TB. In these cases, a purified protein derivative test or an interferon- γ release assay could be helpful due to these tests' high negative predictive value (NPV) (Berbari et al., 2015; Colmenero et al., 2013). Lastly, a parasitic infection – although rare – may be present in some cases but with more unusual pathogens. *Echinococcus* species are parasites with a propensity to infect the bone and cause vertebral hydatid disease (Salaffi et al., 2021).

Among patients in whom targeted investigations, blood cultures, and biopsy cultures are negative, the results of other microbiologic data that correlate with the timing of onset of symptoms, such as preceding urine cultures or known colonization with resistant pathogens, can also be considered when formulating an empiric antimicrobial therapy program (Chenoweth et al., 2018). Transesophageal echocardiography (TEE) may be considered in selected NVO patients to rule out endocarditis as a source of infection (Behmanesh et al., 2019).

## 5 Mimickers of NVO

Some conditions mimic the presentation of NVO. Typical mimickers can be categorized into degenerative, inflammatory, metabolic/deposition, pseudoarthrosis, malignancy, or treatment related, including radiotherapy (Morales, 2018; Salaffi et al., 2021). These conditions are summarized in Table 3. Differentiating NVO from these entities is of utmost importance given the therapeutic and prognostic implications. The role of additional imaging, careful evaluation of images, and histopathology is invaluable in these cases (Morales, 2018). The “claw sign,” seen on diffusion-weighted MRI, was shown to be highly suggestive of Modic type 1 degenerative changes (Patel et al., 2014). In addition, the predominant involvement of one end plate also makes degenerative causes such as Schmorl's nodes more likely than an infectious etiology (Morales, 2018). When considering an inflammatory cause, clues such as multilevel involvement, subluxations, involvement of the posterior elements, and the detection of sacroiliitis would favor the diagnosis of a spondyloarthropathy (Morales, 2018).

**Table 3:** Mimickers of native vertebral osteomyelitis.

Mimickers of NVO		
Pathophysiology	Entity	Differentiators
Degenerative		
	Modic type I changes	Lack of abnormal disc signal or disc hypointensity on T2-weighted MRI
	Schmorl's node	Predominant involvement of only one end plate
	Acute symptomatic calcific discitis	Quick resolution of symptoms and MRI showing a low-signal central focal lesion in the disc
Metabolic		
	CPPD	Pathology results or polarized light microscopy
	Spinal gout	MRI revealing spondylolisthesis, uric acid levels, or surgical sampling of suspected area
	Amyloidosis	MRI revealing a hypointense T2 signal rather than the typical edema-type signal
	Destructive spondyloarthropathy of hemodialysis	MRI revealing severe narrowing of the intervertebral disc spaces, erosions and cystic changes of adjacent vertebral plates, and the absence of significant osteophytosis
Tumor related		
	Metastasis	Preservation of disc space and bone expansion on MRI
	Radiation osteonecrosis	Multiple levels affected with prominent fat replacement above and below the abnormal segment
	Sarcoidosis	Multiple levels involved; confirmed by pathology
Inflammatory		
	Seropositive spondylitis	Pannus formation, multiple levels involved, and possible subluxations
	SAPHO	Characteristic skin manifestations and MRI features
	Spondyloarthridites and Andersson lesions	Location of inflammatory lesions on MRI of the sacroiliac joints and spine
Miscellaneous		
	Pseudoaneurysms	CT scan or conventional angiography

CPPD represents calcium pyrophosphate dihydrate crystal deposition disease, and SAPHO represents synovitis, acne, pustulosis, hyperostosis, osteitis syndrome.

Another example is highlighted in cases of sacral osteomyelitis, where MRI cannot easily distinguish bone remodeling/fibrosis from osteomyelitis, leading to a specificity as low as 22 % despite a high sensitivity. A bone biopsy after debridement is necessary to establish the diagnosis of NVO (Wong et al., 2019). Neuropathic arthropathy (Charcot spine) can also mimic NVO; the presence of exuberant osseous debris on especially CT images can be helpful in establishing this diagnosis.

## 6 New modalities and molecular methods

Novel tools for imaging and microbiologic diagnosis of NVO have emerged. MRI-guided biopsies have long been limited by the resolution offered (often 0.5 T or less). Low-tesla open-magnet MRI scanners have been shown to have an 86 % sensitivity with a 100 % specificity for MRI-guided biopsies (Carrino et al., 2007). Recent advances in MRI have led to even more promising results for these biopsies, owing to the improved resolution and signal-to-noise ratios of modern scanners. However, the efficacy of this method has not been adequately examined, as opposed to CT-guided techniques (Wu et al., 2012).

Novel molecular diagnostic techniques have also garnered significant interest. Studies investigating the use of 16S ribosomal RNA (rRNA) gene polymerase chain reaction (PCR) on suspected cases of NVO have supported its potential role in improving accuracy and time to diagnosis (Sheikh et al., 2017; Choe et al., 2014). These methods complement standard microbiologic methods, particularly difficult to identify microorganisms. Although they lack information on antimicrobial susceptibility, microorganism identification will guide antibiotic therapy (Zimmerli, 2010; Choe et al., 2014; Lecouvet et al., 2004). GeneXpert PCR for spinal tuberculosis is highly sensitive and specific ( > 95 %), with the ability to detect multidrug-resistant tuberculosis (Held et al., 2014).

Metagenomic next-generation sequencing (mNGS) is another novel technique that has proven helpful in identifying various infectious agents. This technology allows the high-throughput sequencing of billions of nucleic acid fragments in a manner much more efficient than the classic Sanger sequencing technique (Lefterova et al., 2015). It carries the benefit of allowing timely detection of one or more pathogens simultaneously, particularly when fastidious, slow-growing or atypical bacteria are implicated (Salipante et al., 2013; Lefterova et al., 2015). Unlike culture methods, mNGS can often determine resistance genes to the molecular levels (Morcrette et al., 2018). The utility of mNGS in osteoarticular infections has been validated in a prospective study conducted on 130 samples of fluid or tissue. The study revealed a positive mNGS rate of 88.5 % compared with 69.2 % associated with culture. However, 16 pathogens isolated in cultures were missed by mNGS in the study due to various reasons. Thus, the technique is only recommended as a complementary study to culture until it is further optimized (Huang et al., 2020). Metagenomic studies are becoming more cost-effective and accurate with time. As reference databases are improved and more pathogen genomes are sequenced, its use is expected to increase and provide more utility, particularly for osteoarticular infections such as NVO (Lefterova et al., 2015; Morcrette et al., 2018).

Many institutions have recently adopted the inoculation of biopsy specimens in blood culture bottles to enhance the recovery of microorganisms. A study using the BACTEC™ 9050 culture bottles (Becton, Dickinson and Company, NJ, USA) for these specimens revealed yields similar to those previously reported in the literature (Pandita et al., 2019). It remains to be seen whether the use of these techniques will optimize the yield of NVO biopsies.

As the methods of NVO diagnosis evolve, early detection continues to be the primary goal. A high index of suspicion can direct a clinician's approach, allowing targeted testing and management. Optimal management of NVO includes accurate identification of the causative agent and treatment with targeted antimicrobial therapy followed by long-term remission. Therefore, we must conduct studies to optimize routinely used techniques, such as image-guided biopsies, and discover new tools such as metagenomic sequencing.

## Data Availability

No data sets were used in this article.
